# Are car safety systems associated with more speeding violations? Evidence from police records in Israel

**DOI:** 10.1371/journal.pone.0286622

**Published:** 2023-08-09

**Authors:** Shani R. Vertlib, Stav Rosenzweig, Ofir D. Rubin, Aviv Steren

**Affiliations:** 1 Department of Business Administration, Guilford Glazer Faculty of Business & Management (GGFBM), Ben-Gurion University of the Negev, Beer-Sheva, Israel; 2 Department of Management, Guilford Glazer Faculty of Business & Management (GGFBM), Ben-Gurion University of the Negev, Beer-Sheva, Israel; 3 Department of Public Policy & Management, Guilford Glazer Faculty of Business & Management (GGFBM), Ben-Gurion University of the Negev, Beer-Sheva, Israel; Tsinghua University, CHINA

## Abstract

Over the past decade, the popularity of installing advanced driver-assistance systems (ADAS) in cars has increased markedly. However, the effectiveness of ADAS is subject to debate, primarily because these systems intervene in drivers’ perceptions and actions and could lead to adaptive behavior. Using complete national data for the installation of three leading safety systems and speeding tickets issued over the course of an entire year, allowed us to pinpoint the impact of these safety systems at a national level. Employing zero-inflated negative binomial regression models, we found that the installation of the three safety systems was associated with higher number of speeding tickets. These findings are in line with the literature that indicates adaptive behavior in the context of risk. However, when we accounted for the proneness to commit other traffic violations, the effect of the safety systems on the prevalence of speeding tickets was evident only for those prone to violations. Further research should be conducted to identify which drivers will be more likely to be affected and under what circumstances and safety system types.

## 1. Introduction

About 1.3 million people are killed in traffic accidents every year, and about 50 million people are injured, with traffic accidents costing about 3% of most countries’ GDP [1]. Among the underlying reasons for traffic accidents and their consequences, the human factor is most important [2]; for example, 94% of traffic accidents in the USA are attributed to drivers’ behavior [3].

Being aware of the potential of advanced technology to impact drivers’ behavior, many researchers and policy makers believe that the most effective strategy to mitigate the occurrence and consequences of traffic accidents is to “provide automatic protection for product and environmental design” [4, p. 82]. Indeed, over the last decade, the popularity of advanced driver-assistance systems (ADAS) installed in cars has increased markedly, and many car manufacturers are currently offering them [5]. However, recent research demonstrates that the effectiveness of ADAS is subject to debate, primarily because they inherently intervene in drivers’ perceptions, actions, and performance [2, 6, 7]. More generally, the literature consistently indicates adaptive behavior in the context of risk. According to the seminal work of Wilde [8], the theory of risk homeostasis argues that individuals strive to counteract any external measures designed to influence the level of their safety by adapting their behavior in a way that would compensate for the change and regain homeostasis that fits their desired level of risk. In light of the literature, the theoretical question remains whether and to what extent human behavior mitigates the advantages of ADAS.

Of the set of driving behaviors, the most meaningful driving behavior–and hence the best researched–is speeding. In 2019 in the USA alone, about 9500 individuals were killed because of speeding, constituting 26% of all traffic fatalities [9]. The importance of speeding is thus widely recognized in the literature, and it is frequently used as a metric for illegal, reckless, and risky driving behavior [10–13].

Against the above background, the present study aims to answer the following research questions: (1) What is the effect of ADAS on the prevalence of risky driving behavior? And (2), in light of the complexity of the effects of ADAS on drivers’ behavior, do these systems differ in their effect on groups that are more prone to violations? Specifically, we examined the behavioral adaptation associated with ADAS at an aggregate level, as expressed by national records of speeding tickets, utilizing parking tickets as a proxy for violation proneness. To address the research questions, we used complete national level data for the installation of safety systems and speeding tickets issued during a particular year—2018.

The present study makes the following contributions to the field: (1) Our use of national level data stands in contrast to the majority of studies assessing the effectiveness of ADAS that use driving simulators and small samples of drivers. (2) Our study also differs from previous studies using aggregate level data that are either limited to safety devices, such as seat belts, helmets, and air bags, or that focus primarily on fatalities and injuries resulting from car accidents. In contrast, our research focuses on speeding—a central behavioral factor leading to car accidents. Speeding is particularly relevant to our research question, because it depends mainly on drivers’ risk-taking behavior (vs. road conditions, the behavior of other drivers, the physical characteristics of the car, and chance; [14].

The rest of the paper is organized as follows. First, we review the literature regarding behavioral adaptation to safety measures. Next, we review recent studies regarding ADAS and the installation of advanced safety systems. We then describe the research context and methods, and finally, we present the results and conclusions.

## 2. Literature review

### 2.1. Behavioral adaptation to safety systems

The complexity of human behavior in response to safety measures is perhaps best exemplified in research dealing with cyclists and helmets, with some studies advocating the importance of helmet wearing and others pointing to some potential negative effects derived from compensating behavior. An examination of previous studies revealed that some found no effect of safety measures on cycling patterns; for example, the study of Schleinitz et al. [15] showed that helmet wearing had no effect on cyclists’ speed. In parallel, a meta-analysis revealed that, in terms of injuries, cyclists have benefited from wearing helmets and there was limited evidence that the helmets had any negative effects [16; see also 17]. In contrast, other studies found that men (but not women) cyclists significantly increased their cycling speed when wearing a helmet, probably because of a compensating behavior [18]. A more recent study testing potential risk-taking behavior found that cyclists reporting always wearing a helmet engaged in risky situations more often than those reporting not wearing a helmet [19]. For motorcyclists, research found that, controlling for risk awareness, motorbike drivers who used helmets tended to compensate for helmet use by speeding [20].

A survey of the literature revealed mixed results for other safety measures as well. For example, one study found that drivers of cars equipped with air bags drove more aggressively and increased the risk of fatalities for others compared with drivers of cars not equipped with airbags [21]. In contrast, another study on airbags concluded that the adverse effect of airbags was the result of self-selection of those installing airbags and that there was no evidence that airbags offset drivers’ behavior [22].

A number of studies on transportation have reported evidence of behavioral adaptation and specifically compensating behavior with regard to voluntary vs. mandatory safety measures. For example, a heated debate evolved around the effectiveness of seat belt laws [e.g., 23–25]. Some studies showed that the severity of injuries did indeed decline with the use of seat belts but the frequency of accidents increased [26], suggesting that regulatory measures, such as mandating seat belt use, have a limited effect in reducing fatalities [27, 28]. Another study found that seat belt laws led to an increase in the number of deaths of both those in the car and those not in the car at the time of the accident, such as pedestrians, cyclists, and motorcyclists [29]. The above studies and others attribute these findings to an offset of the desired results because of a compensating behavior. In contrast, an analysis of motorcycle helmet laws across states in the USA concluded that helmet laws were associated with a significant decrease in injuries [30] and a decrease of 29–33% in fatalities [31]. Regulations pertaining to more advanced safety systems, such as anti-lock brakes (ABS) and electronic stability systems (ESP), were found to have positive externalities that outweigh the cost to the public of these systems, thereby justifying the mandating of these systems [32].

### 2.2. Empirical findings regarding the installation of ADAS

Automatic systems that assist drivers in their driving tasks seem to cause a behavioral adaptation that can unintentionally lead to danger [33]. There are only a few studies dealing specifically with ADAS [e.g., 6, 34]. Shichrur et al. [34] found that older drivers who use ADAS, which includes a collision warning system, drove many more hours and longer distances compared to driving habits prior to the use of the system. Yue et al. [7]–using a Monte Carlo simulation based on predicted endogenous adoption of ADAS–found that the effectiveness of ADAS components could be almost 50% lower than the ideal effectiveness that had previously been proposed. Nonetheless, they estimated that ADAS could prevent 13–30% of crashes, depending on road type and location. Somewhat higher rates of effectiveness of decreasing involvement in crashes were reported by Cicchino [35], who examined data for selected car models equipped with various ADAS systems.

As opposed to examining end results in terms of crashes, Yu, B. et al. [6] examined hard breaking driving behavior and found no evidence that ADAS changed the likelihood of this behavior. Similarly, a simulator study by Jokinen and Kujala [36] found that the installation of ADAS led to a behavioral adaptation that increased risky driving. In examining a survey of ADAS users, McDonald et al. [37] found that the survey participants had only limited knowledge about the capabilities of ADAS and demonstrated a considerable over-reliance on those systems. In line with the findings of Yue et al. [7], research suggested that the driving context and environment are critical, because these are the main factor affecting drivers’ decision making in ADAS-equipped vehicles [5, 38].

The main contribution of our study is that it is based on aggregate national data that enables us to examine how real-world ADAS installation and speeding tickets are interrelated. These aggregate data enabled us to assess the macro-level behavioral consequences of the installation of the examined safety systems. To the best of our knowledge, this is the first study to examine the effectiveness of ADAS at a national level.

## 3. Research context and data

Israel has no local car industry, and all cars are imported. Moreover, economically speaking, Israel is an island. Buying a car outside the country is effectively impossible due to minimal trading relationships with neighboring countries and the long bureaucratic processes required for private imports or for crossing one of Israel’s borders by car. Because of these special characteristics, all cars in Israel are closely monitored, as are all traffic tickets.

**Fig 1:**
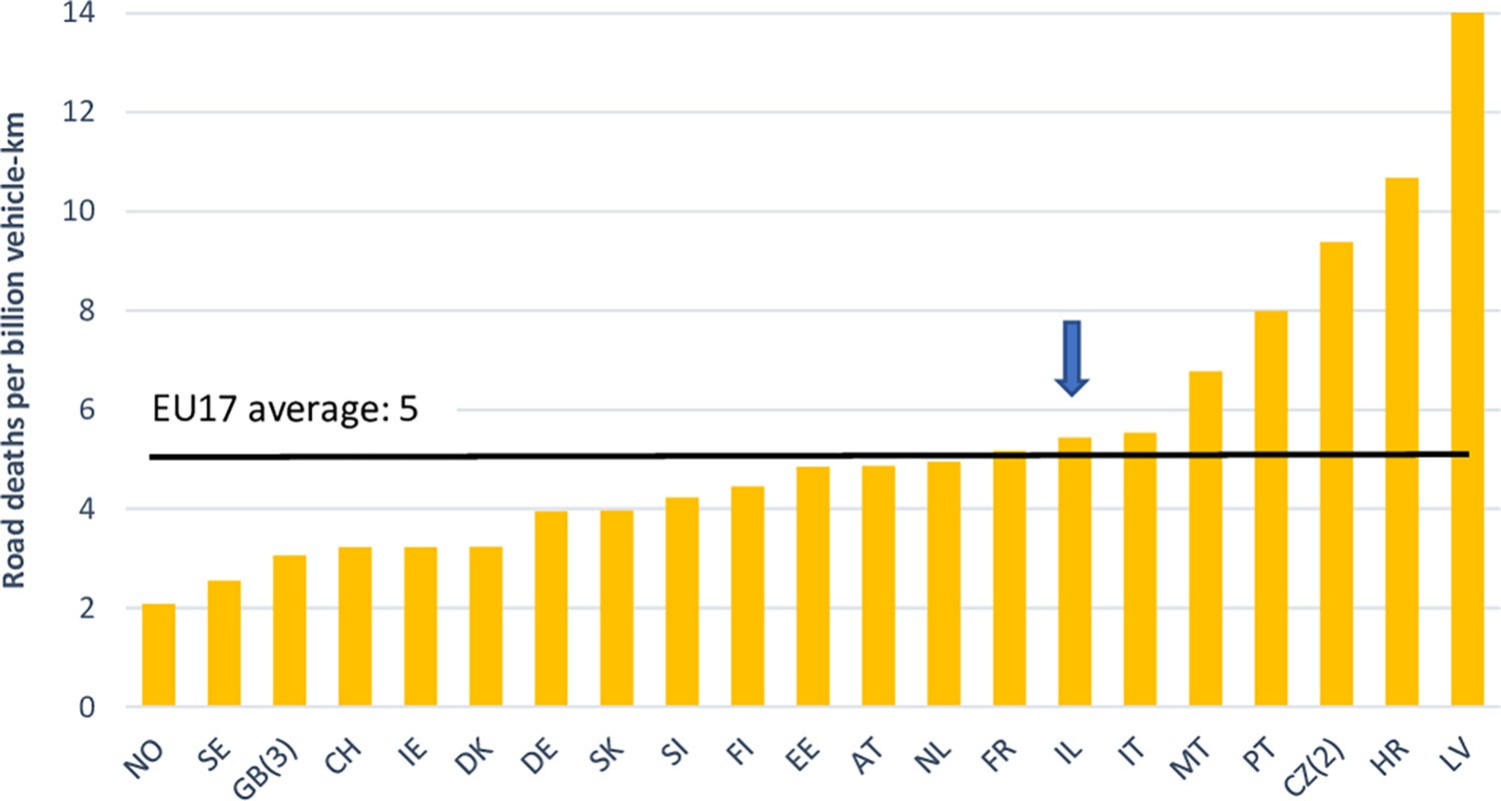
Road deaths per billion vehicles-km, 2019–2021 average, Israel (IL) vs. other European countries. Source: European Transport Safety Control (ETSC), 2022, Ranking EU progress on road safety, 16^th^ road safety Performance Index Report, June 2022, p. 19.

According to the European Transport Safety Control (ETSC) most recent report, the number of deaths per million inhabitants in Israel in 2021 was 38.5, in the mid-range of European countries, and slightly below the EU average (44.6). The 3-year average number of deaths per billion vehicle-kilometers in Israel was 5.44, similar to that of the EU of 5.12 (Fig 1; [39]). About 250K new private cars are sold in Israel each year (Authors’ calculations based on data obtained from the Central Bureau of Statistics, 2021). In an attempt to mitigate the damages of traffic accidents, the Israel Government supports the installation of safety systems in new cars.

We examined all speeding tickets recorded by the Israel Police in 2018. We matched this data with the records of the Israel Ministry of Transportation and Road Safety for all cars active in 2018. Specifically, we analyzed a traffic census of all speeding tickets attributed to specific car models using the road system in Israel. Because we have complete records of all speeding tickets in the country throughout the year, we are able to capture a reflection of the true effects, which already include the influence of weather conditions during the year of investigation. In our analysis, each observation pertained to a specific car model in terms of brand, engine size, engine type, horsepower, safety systems, etc., and the number of speeding tickets that drivers of this specific car model received in 2018. Data regarding specific rare models of which less than 20 cars were sold was not made available by the police due to privacy considerations (less than 5% of the car population). Overall, our data included 2,620,123 active cars categorized by the Ministry of Transportation into 6,318 unique car models.

## 4. Method

### 4.1. Model

The population of car models can be divided into two groups: one whose drivers are not prone to violations, i.e., drivers of car models who do not commit speeding violations, and the other whose drivers are prone to committing speeding violations. This distribution is therefore characterized by excess zeros. Previous studies generally suggest that data characterized by excess zeros could result in biased parameter estimations and create a poor model fit [40, 41]. Excess zeros may reflect incomplete data, a small sample size, a short observation period, and limited spatial scale, all of which could cause bias due to unobserved heterogenous factors. To overcome the problem of incomplete data and the resulting bias, techniques such as generating synthetic crash data, machine learning, artificial intelligence, random parameter techniques, or a mixture of methods are often used [42]. Alternatively, when complete national data with respect to time and space are used, the excess zeroes reflect a true phenomenon as the entire range of occurrences over space and time is recorded. Such is the case of the present study which uses census data of all vehicles in Israel.

We analyzed the data by using a zero-inflated negative binomial (ZINB) regression model. This model is often used to examine real-life count data that is characterized by overdispersion and excess zeros [e.g., 43–45]. We report overdispersion tests in the Results section.

The probability that the binary process results in a zero outcome is 0≤*p*≤1, and (1−*p*) is assigned to the outcomes that follow a negative binomial distribution *f*(*y*). The ZINB distribution function for committing a speeding violation *Y*_*i*_ is written as:

$$P(Y_{i}=y_{i})=\left\{ \begin{array}{cc} p_{i}+(1-p_{i})f(y) & y_{i}=0\\ \left(1-p_{i})f(y\right) & y_{i}=1,2,\ldots \end{array}\right.$$

where the negative binomial distribution is given by:

$$f(y)=P(Y_{i}=y_{i})=\frac{\Gamma (y_{i}+\alpha^{-1})}{\Gamma (\alpha^{-1})\Gamma (y_{i}+1)}{\left(\frac{1}{1+\alpha \mu_{i}}\right)}^{\alpha^{-1}}{\left(\frac{\alpha \mu_{i}}{1+\alpha \mu_{i}}\right)}^{y_{i}}$$

The parameter *μ*_*i*_≥0 represents the average number of speeding tickets per car model, *α*>0 is the dispersion parameter, and Γ(∙) is the gamma function.

We assume a logistic link function given by $p_{i}=\frac{e^{\gamma_{1}z_{1i}+\gamma_{2}z_{2i}}}{1+e^{\gamma_{1}z_{1i}+\gamma_{2}z_{2i}}}$, where *γ*_1_ estimates the constant term, and *γ*_2_ is the coefficient of *z*_*i*_, which is the number of parking tickets received by car model *i* = 1,…,*n*. This assumption is based on previous findings demonstrating that drivers most likely to commit violations and to be involved in traffic accidents are less likely to observe transportation regulations [e.g., 28, 46]. Indeed, the correlation between these variables in our data is r = 0.914 (*p*<0.01). Finally, we specify a regression model with *k* regressors (including a constant term) as follows:

$$\mu_{i}=e^{\beta_{1}x_{1i}}+e^{\beta_{2}x_{2i}}+\cdots +e^{\beta_{k}x_{ki}}$$

where the *x*_1_…*x*_*k*_ each include a safety system as an independent variable, control variables, fixed effects and interaction terms, as detailed below.

### 4.2. Measures

#### 4.2.1. Dependent variable

**Fig 2:**
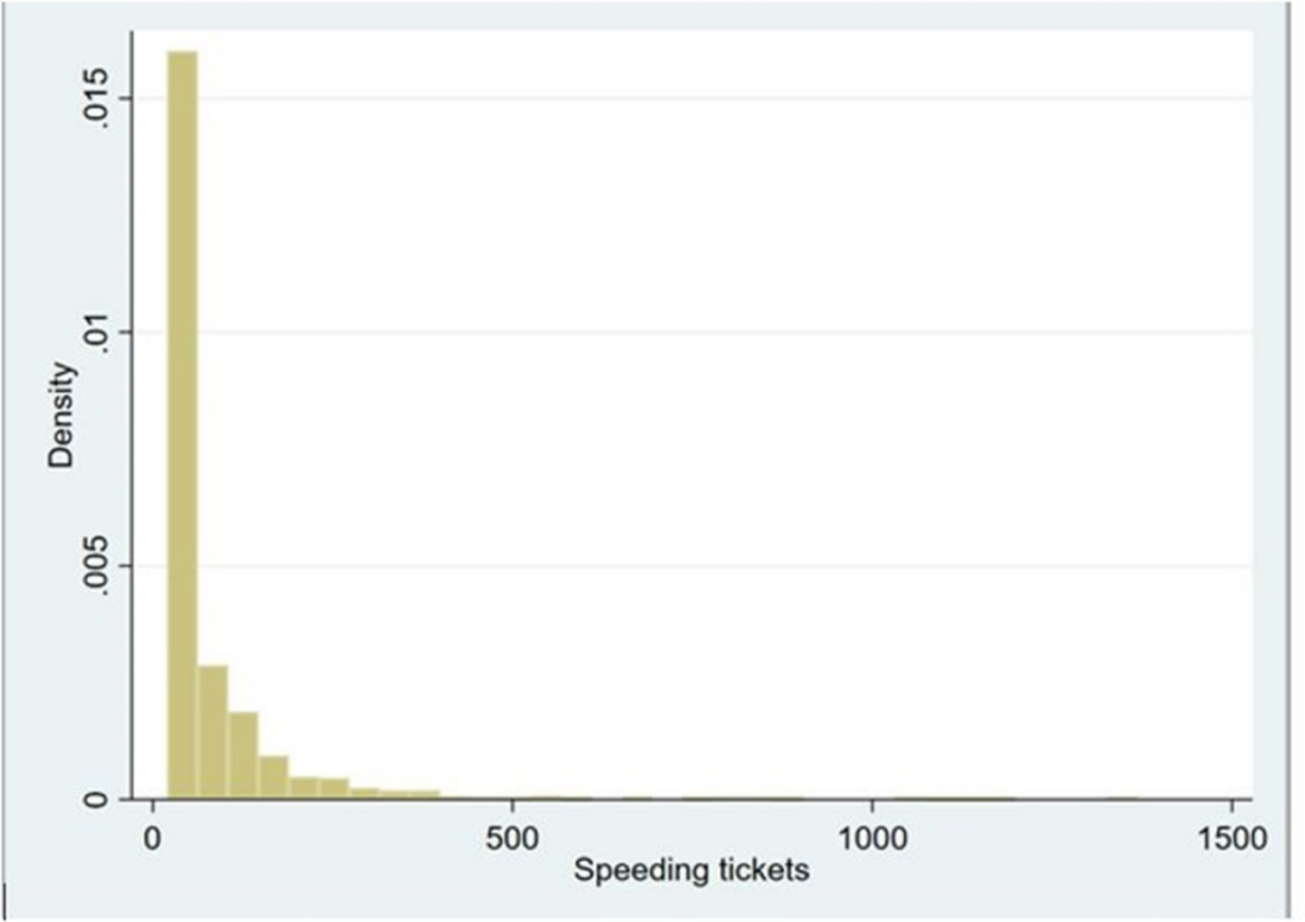
Distribution of speeding tickets across car models in 2018 (n = 1,622).

Speeding is the traffic violation most commonly associated with reckless driving and is thus typically used in studying the behavior of drivers [e.g., 47, 48]. Moreover, it is specifically connected with adaptive behavior [12, 49]. We analyzed the number of speeding tickets associated with each specific car model in the study year. Not all traffic violations are observed and enforced by the police, but we have no reason to suspect biased enforcement. In the traffic census data that we used, there were no speeding tickets associated with most, i.e., 75%, of the specific car models. The remaining 25% are presented in Fig 2, which shows the distribution of speeding tickets across car models. The excess zeros in the dependent variable requires an adequate estimation, as we detail in section 4.1.

#### 4.2.2. Independent variables

Safety systems (versus the lack thereof) constituted our independent variables. Specifically, we examined three safety systems that alert the driver in dangerous situations—deviation control, front distance control, and pedestrian identification control. Deviation control is a system that alerts the driver when the car deviates from the lane without signaling. Front distance control alerts the driver as to the danger of a rear-end collision with the vehicle ahead. Pedestrian identification control alerts the driver when a pedestrian or a cyclist is in close proximity. All three systems provide audio and visual alerts during potentially risky situations.

#### 4.2.3. Control variables

For each specific car model, we controlled for the number of active cars and the average number of kilometers traveled by these cars. Because drivers of different car categories may exhibit different characteristics in terms of spatial, temporal, and behavioral driving patterns [47], we controlled for car category (family cars, SUVs, sports, mini, luxury, executive, minivans, and commercial). All this data was obtained from the Ministry of Transportation and Road Safety. We also included data on parking tickets, as we elaborate in the section 4.1.

#### 4.2.4. Descriptive statistics

Table 1 presents descriptive statistics per specific car model (n = 6,318). The total number of cars in the private car fleet in Israel in the study year was 2,620,123, and each of these cars traveled an average of 20,248 km in that year. The average number of cars per model was 414.7, with the most popular specific car model (31,080 units) being the Mazda 3, with a 1598-cc gasoline engine, an automatic gearbox, and a horse power of 105. During the study year, the police issued 132,901 speeding tickets and 77,436 parking tickets to the cars in the analysis. The average number of speeding tickets per car model in the investigated year was 21.04, which was approximately 0.051 tickets per car.

**Table 1:** Descriptive statistics (n = 6,318).

Variable	Mean	Max	Min	SD
Speeding tickets	21.035	1,368	0	71.830
Parking tickets	12.256	1,425	0	48.008
Average kilometers traveled	20,248	92,967	3.49	12,095
Number of cars	414.71	31,080	20	1147.71
Deviation control	0.295	1	0	0.450
Front distance control	0.319	1	0	0.466
Pedestrian identification control	0.233	1	0	0.423
Family car	0.270	1	0	0.444
Minivan car	0.055	1	0	0.228
SUV car	0.275	1	0	0.446
Luxury car	0.042	1	0	0.200
Executive car	0.106	1	0	0.308
Sports car	0.019	1	0	0.138
Mini car	0.205	1	0	0.404
Commercial vehicle	0.025	1	0	0.156

With regard to the safety systems, the adoption rates were 29.5% for deviation control, 31.9% for front distance control, and 23.3% for pedestrian identification control. The two car categories with the highest number of specific car models were SUVs (27.5%) and family cars (27%). These were followed by mini cars (20.5%), executive cars (10.6%) and minivan, luxury, commercial vehicles and sports cars (14.4% in total).

## 5. Results

We begin our estimation strategy by presenting the results of the main research question: is the presence of safety systems associated with speeding tickets. We then introduce a parking tickets variable, which serves as a proxy of proneness to committing traffic violations, as well as an interaction term between parking tickets and the installation of safety systems to test the relevance of the safety systems to a specific group, those who are prone to traffic violations.

Table 2 presents the results of the ZINB regression model estimation examining the association between safety systems and speeding tickets, where the level of analysis is the distinct car model. The estimation of the coefficient of ln(*α*) is significant across the three estimated models (p < 0.001), indicating overdispersion of the dependent variable and therefore the suitability of a zero-inflated negative binomial model over a zero-inflated Poisson model. Nevertheless, to make sure that our findings do not depend upon the overdispersion assumption, we estimated a zero-inflated Poisson model. The results remain similar.

**Table 2:** Model estimation–predicting speeding tickets (standard errors in parentheses).

Dependent variable:	(1)	(2)	(3)
Speeding tickets	Deviation control	Front distance control	Pedestrian identification control
Safety system	0.101 (0.032)***	0.115 (0.029)***	0.128 (0.030)***
Average kilometers traveled	7.33e-06 (1.361e-06)***	7.03e-06 (1.371e-06)***	7.35e-06 (1.369e-06)***
Number of cars	0.0004 (9.699e-06)***	0.0004 (9.706e-06)***	0.0004 (9.715e-06)***
Car category fixed effects	**✓**	**✓**	**✓**
Constant	3.573 (0.045)***	3.576 (0.045)***	3.573 (0.045)***
Inflate			
Parking tickets	-0.161 (0.006)***	-0.161 (0.006)***	-0.161 (0.006)***
Constant	2.074 (0.044)***	2.074 (0.044)***	2.074 (0.044)***
ln (*α*)	-1.345 (0.036)***	-1.347 (0.036)***	-1.348 (0.036)***
*α*	0.261 (0.009)	0.260 (0.009)	0.260 (0.009)
Observations	6,318	6,318	6,318

*** p < 0.001

The coefficient of parking tickets, which we use as the zero-inflation variable, is negative and significant across all three safety systems (*p* < 0.01). This result suggests that specific car models prone to commit parking violations are also prone to commit speeding violations. The parking violations variable, therefore, provides a sound basis for the identification of those specific car models whose drivers are prone to receiving tickets.

The association between the safety systems and speeding tickets is positive across all three safety systems (deviation control: *β* = 0.10, front distance control: *β* = 0.12, pedestrian identification control: *β* = 0.13, *p* < 0.001). These results suggest that car models with these safety systems are associated with a higher number of speeding tickets, in accordance with the body of literature indicating behavioral adaptation. Prior studies have also reported negative consequences of the utilization of ADAS. Naujoks and Totzke [50] reported that congestion tail warning systems might limit the positive effect of these systems through behavioral adaptation which is manifested, among other things, through over-speeding. Naujoks et al. [51] reported an adverse drivers’ response to false or unnecessary alarms of cooperative advisory warning systems, where behavioral adaptation limits drivers’ compliance to these alarms. Similarly, Bao et al. [52] find that ADAS reduced pedal control of teen drivers. However, Yu, H. et al. [42], found no association between ADAS and hard breaking behavior across age groups.

The average number of kilometers travelled and the number of active cars, which control for the road-presence of car models, are positively associated with speeding tickets, as expected [e.g., 42]. For example, the coefficient of average number of kilometers travelled is in line with prior research suggesting an association between mileage and speeding tickets [46].

Next, we include the number of parking tickets as an explanatory variable and introduce an interaction term between parking tickets and safety systems. This interaction term represents the tendency of drivers prone to committing parking violations and have one of the safety systems to commit speeding violation.

Table 3 presents the results of the regression estimation including the interaction term. We find that the association between the safety systems and speeding tickets is not statistically significant across all three regression models (*p* > 0.1). This result is in line with Bao et al. [52], who reported that ADAS do not reduce tailgating or speeding behavior. As expected, the coefficient of parking tickets is consistently positive and significant (*β* between 0.0073 and 0.0074, *p* < 0.001). This finding is in line with Pan et al. [53], who reported that drivers prone to risky behavior were more likely to exhibit poor speed control. Interestingly, the group of car models with parking tickets and safety systems (represented by the interaction term) is associated with more speeding tickets compared with the other car models (*p* < 0.001). These results indicate that, overall, those drivers prone to violations are even more prone to risky driving in the presence of safety systems. These findings are consistent across all three safety systems.

**Table 3:** Model estimation–predicting speeding tickets (standard errors in parentheses).

Dependent variable:	(1)	(2)	(3)
Speeding tickets	Deviation control	Front distance control	Pedestrian identification control
Safety system	-0.0023 (0.036)	-0.015 (0.035)	-0.39 (0.038)
Average kilometers traveled	2.5e-06* (1.3e-06)	1.9e-06 (1.3e-06)	2.7e-06** (1.3e-06)
Number of cars	5.6e-05** (2e-05)	5.1e-05* (2e-05)	5.6e-05*** (2e-05)
Parking tickets	0.0073*** (5.1e-04)	0.0073*** (5e-04)	0.0074*** (5e-04)
**Parking tickets × safety system**	0.0040*** **(5.5e-04)**	0.0043*** **(5.5e-04)**	0.0036*** (5.8e-04)
Car category fixed effects	**✓**	**✓**	**✓**
Constant	3.69*** (0.042)	3.7*** (0.041)	3.68*** (0.042)
Inflate			
Parking tickets	-0.16*** (0.005)	-0.16*** (0.005)	-0.16*** (0.005)
Constant	2.07*** (0.044)	2.07*** (0.044)	2.07*** (0.044)
ln (*α*)	-1.526 (0.036)***	-1.536 (0.036)***	-1.518 (0.036)***
*α*	0.217 (0.008)	0.215 (0.008)	0.219 (0.008)
Observations	6,318	6,318	6,318

*** p < 0.001

** p < 0.01

* p < 0.05

## 6. Discussion and conclusion

The present study takes advantage of complete national level data for the installation of car safety systems and speeding tickets during one year. Because we examined a single year, enforcement, infrastructure, and economic terms were consistent across all observations. This allowed us to pinpoint the impact of three safety systems at a national level.

We found that disregarding the proneness to traffic violations, the examined safety systems demonstrate a positive association with the prevalence of speeding tickets. These findings are in line with prior literature suggesting adaptive behavior in the presence of safety systems [e.g., 6, 36]. When we focused on proneness to traffic violations by taking the parking tickets into account, the effect of the safety systems on the prevalence of speeding tickets was correlated with speeding only for observations associated with parking tickets.

The literature consistently indicates adaptive behavior in the context of risk. According to the theory of risk homeostasis, individuals strive to counteract any external measures designed to influence the level their safety. This view argues that “the sum of sins is constant” [8, p. 224], i.e., any measure to alleviate risk will result in adaptive behavior that will reinstate the former level of risk. Our findings suggest that behavioral characteristics make a difference: for those prone to violations, we are able to observe accentuated adaptive behavior, whereas we are unable to observe this adaptive behavior for the rest of the population.

Following prior research that examines specific populations that could be prone to aggressive driving and excessive violations [e.g., 54, 55], we zoom-in on drivers of car models associated with traffic violations. Following studies that connect parking violations with other immoral behaviors [e.g., 56], we identify a group of potential violators as those committing parking violations. The high correlation between parking tickets and speeding tickets (*r* = 0.914, *p* < 0.01) attests that the theoretical identification is supported by empirical findings. Importantly, drivers prone to (parking) violations are even more prone to speeding given they have installed safety systems. These results indicate that the installation of safety systems does not always work in the expected and desired way. Further research should be conducted to identify which drivers will be more likely to be affected and under what circumstances and safety system types.

In light of the findings in the present study, when decision makers design a policy that incentivizes or mandates the installation of safety systems, they should consider the possibility that adaptive behavior will be accentuated, thereby potentially offsetting benefits. There is an ongoing debate in the literature, on whether the installation of safety measures should be recommended or mandated [e.g., 16]. Future research can examine the effect of regulating the installation of ADAS on different types of drivers in terms of risk preferences, especially given that prior research found ADAS to interact with sensitivity and reaction time [57].

The present study has some limitations. First, our unit of analysis is the specific car model, since both the Israel Police and the Israel Ministry of Transportation and Road Safety report traffic violations and systems installation at the specific car model level. Presumably, driver level data would have some advantages over model data in terms of associating individuals with specific behaviors. Moreover, driver level data could include variables regarding specific driver, car, road, traffic, and environment characteristics. Importantly, addressing unobserved heterogeneity is critical especially when analyzing crashes, because the complete circumstances that could affect a specific incident are not usually recorded, which could result in serious specification problems and bias parameter estimates [58]. For example, Guo et al. [59] report that cyclists’ crashes have a positive association with traffic exposure variables and a negative association with cycling network indicators. Guo et al. [60] find that while many factors affect cyclists’ red light running behavior, factors associated with unobserved heterogeneity include gender, bicycle type, signal type, separation width and bicycle volume. Finally, Guo et al. [61] examine the probabilities of crashes across three collision types suggest that each collision type is associated with different risk factors.

Nonetheless, the level of analysis in the present study has two meaningful advantages: It facilitates a focus on the effect of technology that is above and beyond any context or individual characteristics, and it provides an aggregate level perspective that is currently missing in research regarding the overall effectiveness of ADAS. Second, the present study focuses on speeding violations, however this is only one of a variety of potential measures of risk taking and driving behavior. Future research can examine other traffic violations such as disobeying traffic signs, lane deviation, etc., as these might have a different expression among different types of drivers, and their examination could provide a broader view of the connection between the installation of safety systems and risky driving behavior.

In light of the findings in the present study, when decision makers design a policy that incentivizes or mandates the installation of safety systems, they should consider the possibility that adaptive behavior will be accentuated, thereby potentially offsetting benefits.
